# Dynamic Conformational Changes in MUNC18 Prevent Syntaxin Binding

**DOI:** 10.1371/journal.pcbi.1001097

**Published:** 2011-03-03

**Authors:** Dana Bar-On, Esther Nachliel, Menachem Gutman, Uri Ashery

**Affiliations:** 1 Department of Neurobiology, Tel Aviv University, Tel Aviv, Israel; 2 Laser Laboratory for Fast Reactions in Biology, Department of Biochemistry, George S. Wise Faculty of Life Sciences, Tel Aviv University, Tel Aviv, Israel; National Cancer Institute, United States of America and Tel Aviv University, Israel

## Abstract

The Sec1/munc18 protein family is essential for vesicle fusion in eukaryotic cells via binding to SNARE proteins. Protein kinase C modulates these interactions by phosphorylating munc18a thereby reducing its affinity to one of the central SNARE members, syntaxin-1a. The established hypothesis is that the reduced affinity of the phosphorylated munc18a to syntaxin-1a is a result of local electrostatic repulsion between the two proteins, which interferes with their compatibility. The current study challenges this paradigm and offers a novel mechanistic explanation by revealing a syntaxin-non-binding conformation of munc18a that is induced by the phosphomimetic mutations. In the present study, using molecular dynamics simulations, we explored the dynamics of the wild-type munc18a versus phosphomimetic mutant munc18a. We focused on the structural changes that occur in the cavity between domains 3a and 1, which serves as the main syntaxin-binding site. The results of the simulations suggest that the free wild-type munc18a exhibits a dynamic equilibrium between several conformations differing in the size of its cavity (the main syntaxin-binding site). The flexibility of the cavity's size might facilitate the binding or unbinding of syntaxin. *In silico* insertion of phosphomimetic mutations into the munc18a structure induces the formation of a conformation where the syntaxin-binding area is rigid and blocked as a result of interactions between residues located on both sides of the cavity. Therefore, we suggest that the reduced affinity of the phosphomimetic mutant/phosphorylated munc18a is a result of the closed-cavity conformation, which makes syntaxin binding energetically and sterically unfavorable. The current study demonstrates the potential of phosphoryalation, an essential biological process, to serve as a driving force for dramatic conformational changes of proteins modulating their affinity to target proteins.

## Author Summary

Protein phosphorylation plays a significant regulatory role in multi-component systems engaged in signal transduction or coordination of cellular processes, by activating or deactivating proteins. The potential of phosphorylation to induce substantial conformational changes in proteins, thereby changing their affinity to target proteins, has already been shown but the dynamics of the process is not fully elucidated. In the present study, we investigated, by molecular dynamics simulations, the dynamic conformational changes in munc18a, a protein that is crucial for neurotransmitter release and interacts tightly with the SNARE syntaxin-1. We further investigated the conformational changes that occur in munc18a when it is phosphorylated, reducing its affinity to syntaxin-1a. The results of the simulations suggest that there is a conformational flexibility of the syntaxin-unbounded munc18a that allows changes in the shape of the syntaxin-1a binding cavity. *In silico* insertion of phosphomimetic mutations into munc18a led to a reduction in the flexibility and closure of the syntaxin-binding site. We suggest that the reduced affinity of phosphorylated munc18a to syntaxin-1a stems from the difficulty of syntaxin-1a to bind to the munc18a closed-cavity conformation, induced by the PKC phosphorylation of munc18a.

## Introduction

Intracellular membrane fusion in eukaryotes is mediated by a well-conserved fusion machinery composed of SNARE (soluble N-ethylmaleimide-sensitive factor attachment protein receptor) and SM (Sec1/munc18-like) proteins [1]. In the early studies, munc18a was shown to bind syntaxin, one of the central SNARE members and block ternary SNARE complex formation, suggesting that it plays a negative regulatory role [2], [3]. However, genetic and biochemical studies indicated that SM proteins play a positive essential role as demonstrated by their null mutants; studies with mutated worms, flies and mice lacking munc18a, revealed a dramatic decrease in secretory granule fusion, docking and priming [4], [5], [6]. Therefore, the central hypothesis, to date, is that SM proteins play several roles depending on their mode of binding to the SNARE members [7], [8]. The first mode of interaction that was discovered [9] relates to the binding of munc18a to a stable closed-conformation of syntaxin. This mode of interaction allows the specific transfer of syntaxin through the endoplasmic reticulum and the Golgi apparatus to the plasma membrane, keeping syntaxin from engaging to ectopic intracellular SNARE complexes [10], [11].

Recent studies demonstrate that SM proteins bind only or additionally to a short peptide present at the N-terminus of syntaxin, designated as the N-peptide [1], [10], [12]. This mode of interaction was intensively investigated in the last few years and its importance is under a strong debate. One of the main hypotheses for the role of the interaction of munc18a with the N-terminal of syntaxin is that this interaction allows munc18a to bind the SNARE ternary complex suggesting a stimulatory role for munc18a in the last stages of SNARE-mediated fusion [13].

**Figure 1:**
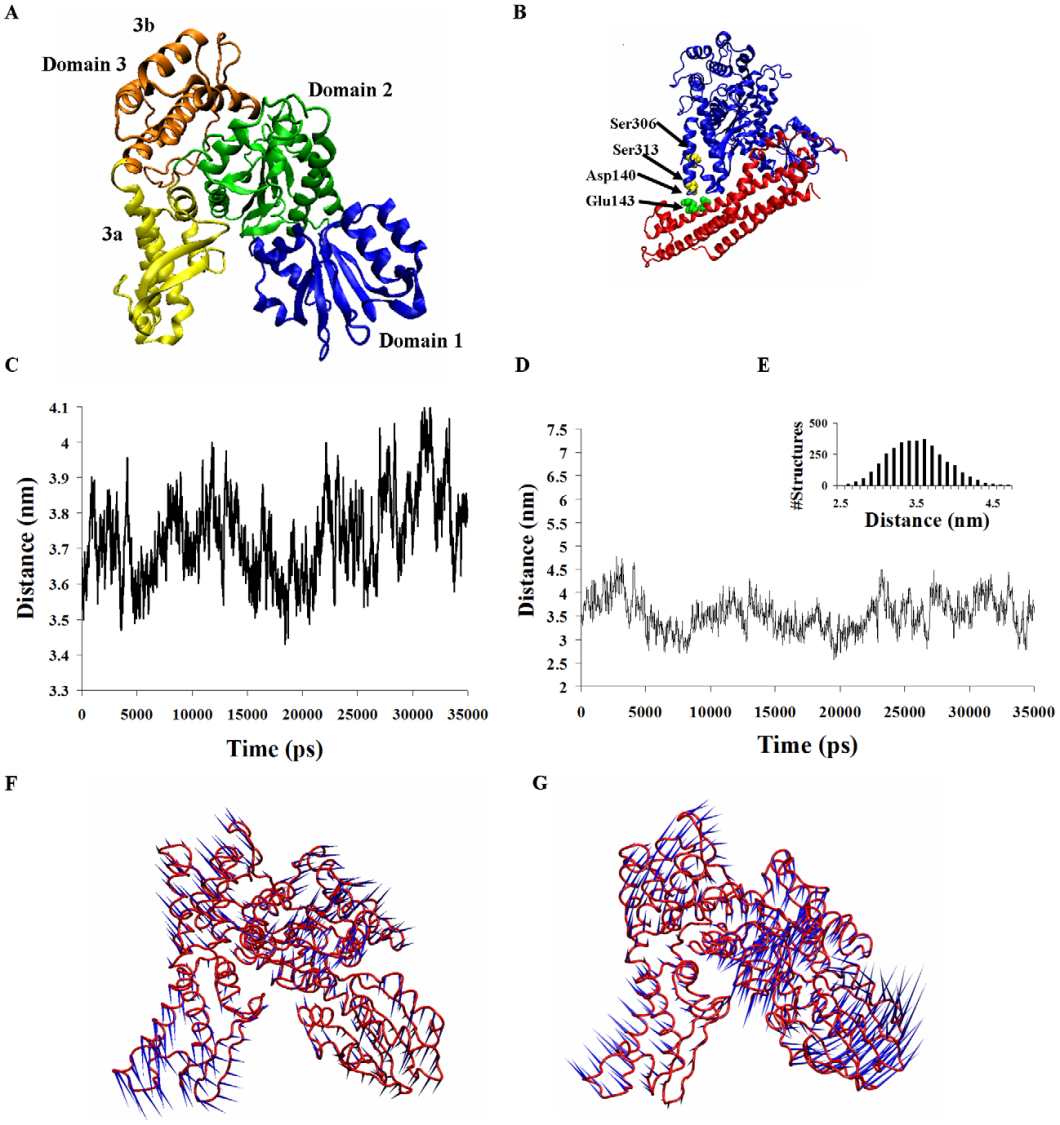
Dynamic equilibrium between several open- and closed-cavity conformations of wild-type munc18a. A) Structure of munc18a (3C98.pdb). B) Ribbon presentation of syntaxin-1a-munc18a complex including the location of the munc18a phosphorylation sites (Ser 306 and Ser 313) and adjacent residues of syntaxin-1a (Asp 140 and Glu 143). C) Time-dependent change in the distance between the centers of mass of domains 1 and 3a during the wild-type munc18a simulation (simulation 1). D) The distance between residues Gly 26 and Glu 273. E) Histogram of the distribution of the distance between Gly 26 and Glu 273. F–G) Porcupine plots based on ED analysis of two of the main motion vectors (the first and the fourth) of munc18a wild-type; the direction and the length of the ‘needles’ in blue indicate the direction and extent of the motion respectively. Closure motion of the cavity (F) opening motion of the cavity (G).

The rat munc18a, which was structurally resolved as part of the complex with syntaxin-1a [9], [12], is an arched-shaped three-domain protein (Figure 1A) that embraces syntaxin in a cavity located between domains 3a and 1 (Figure 1, A and B). Phosphorylation by protein kinase C (PKC) or phosphomimetic mutations in residues 306 and 313 (S306D, S313D) of munc18a modulate this interaction by reducing the affinity to form a complex [14], [15]. Previous studies have suggested that replacement of the polar serine moieties in domain 3a of munc18a by phosphate groups or negatively charged glutamates disrupts the complex due to electrostatic repulsion between munc18a and the adjacent area of syntaxin (Figure 1B), which contains acidic residues [14].

In the present study, munc18a dynamics was studied, for the first time, using molecular dynamics (MD) simulations under different conditions for several hundred nanoseconds. We show that in the absence of syntaxin, wild-type munc18a exhibits a dynamic equilibrium between several states, differing in the size of the syntaxin-binding site (the cavity between domains 3a and 1). In the next step, we examined the dynamic behavior of the phosphomimetic munc18a^S306D,S313D^ and we show that following *in-silico* insertion of the mutations into the wild-type structure, munc18a adopted a rigid closed-cavity conformation which makes syntaxin binding less probable. The closed-cavity conformation is induced specifically by the PKC phosphomimetic mutations and reversible upon dephosphorylation of the protein back to the wild-type form.

## Results

### Structural fluctuations of the syntaxin-binding site of munc18a

In the present study, munc18a dynamics was studied using molecular dynamics (MD) simulations, a powerful method in which the dynamics and conformational changes in proteins can be followed in a virtual fashion. We performed three MD simulations of the wild-type munc18a (termed 1, 2 and 3, Table 1) as described in details in the Methods section. The simulations were performed under the same conditions; accept for applying two distinct informatics tools; Swiss-Pdb [16] or Rosetta [17], [18], [19] for *in silico* reconstruction and structural modeling prediction of regions in the protein that were not resolved in the crystal structure [9].

**Table 1:** Summary of details concerning the simulations of the wild-type and phosphomimetic mutant (munc18a^S306D,S313D^) munc18a.

munc18a wild type					
Simulation	Simulation description	Duration (ns)	Net charge of munc18a	Number of Water molecules	Number of ions
1	Swiss-PDB tool was used for completion of missing regions	35	−4e	35,097	74 Na^+^, 70 Cl^−^
2	Swiss-PDB tool was used for completion of missing regions	35	−4e	35,097	74 Na^+^, 70 Cl^−^
3	Rosetta suite was used for completion of missing regions	35	−4e	34,972	73 Na^+^, 69 Cl^−^

munc18a^S306D, S313D^					
M1	The mutations were inserted into the structure by replacing the two serine residues in the crystal structure of the protein with two glutamates	∼37	−6e	35,092	76 Na^+^, 70 Cl^−^
M2	The mutations were inserted into the structure by replacing the two serine residues in the final munc18a structure of simulation 1 (t = 35 ns) with two glutamates	35	−6e	37,983	81 Na^+^, 75 Cl^−^

The high resemblance of the basic dynamics characteristics of munc18a in the three simulations (Text S1 and Figures S1 and S2) allowed us to evaluate the general relative inter-domain motions of the protein and attribute them to the activity and function of the protein. We monitored specifically the changes in the main syntaxin-binding site of munc18a, which is the area of the cavity between domains 3a and 1 [9]. We first measured the change in the distance between the centers of mass of domains 3a and 1 (Figure 1C) and of the distance between specific residues (Gly 26 in domain 1 and Glu273 in domain 3a) on both sides of the cavity (Figure 1, D–E) during the simulations. The measurements showed that the distances frequently change, indicating structural fluctuations of these domains and dynamic changes in the size of the cavity, becoming wider or narrower (Figure 1, C–E).

During the simulations, the main motions of the protein were isolated from its overall movement using an essential dynamics (ED) analysis. ED analysis is a method for isolating the various modes of motion of a protein during the simulation by yielding a set of eigenvectors corresponding to its internal motions namely the amplitudes and the directions of the motions [20]. The vectors are scaled according to the time scale of the motion from the slowest undulations which generally correspond with motions of large regions in the protein, and up to the fast and high-frequency local fluctuations.

The ED analysis of the wild-type munc18a simulations clearly illustrated that the main motion vectors exhibit opening and closure of the cavity between domains 3a and 1 (Figure 1, F–G) dramatically changing its size. The high flexibility in the size of the munc18a cavity probably assists in binding or unbinding of syntaxin or other target proteins that bind munc18a in other regions as well (such as CDK5 for example) [9], [21].

### The dynamics of free wild-type munc18a resembles the crystal structures of its homolog, sSec1

**Figure 2:**
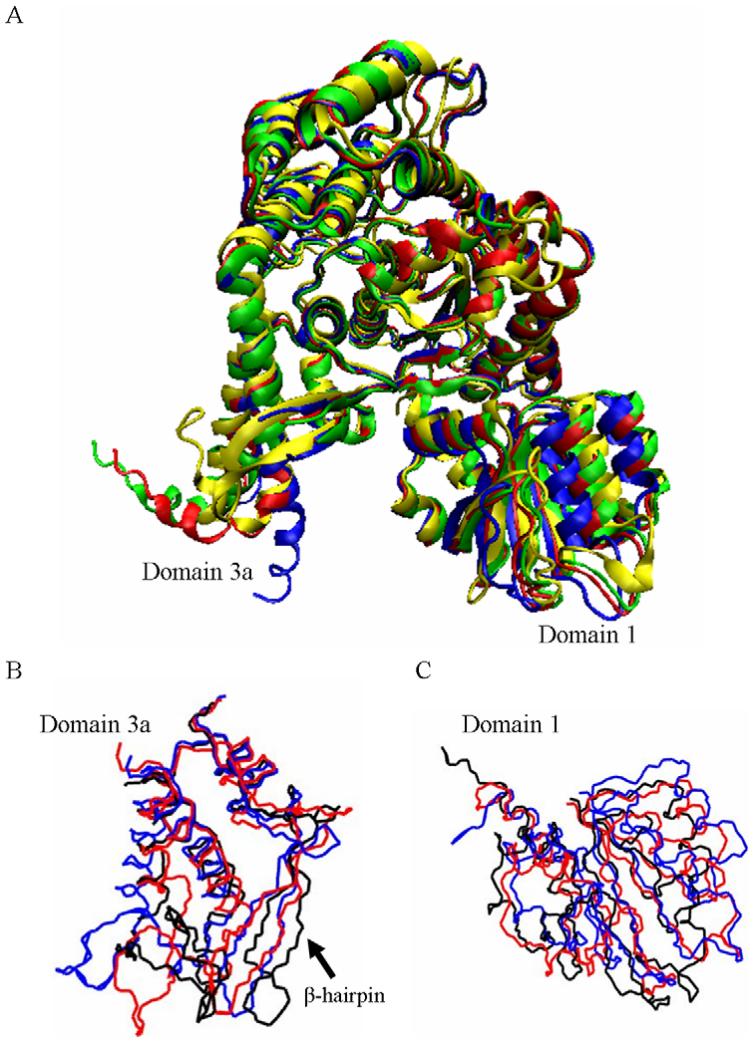
Comparison of free munc18a dynamics to its squid homologs' crystal structures. A) Superposition of the backbone of four crystal structures presented in ribbon form; green, blue and red: the three resolved crystal structures of the squid sSec1: green (1EPU.pdb), blue (1FVF.pdb) and red (1FVH.pdb); yellow: the crystal structure of munc18a taken from the complex with syntaxin ([12], 3C98.pdb). B) Snapshots of domain 3a taken from the last frame of the first eigenvector in each of the three wild-type simulations; blue: 1, red: 2, black: 3. C) Snapshots of domain 1 taken from the last frame of the main eigenvectors of each of the three MD simulations; blue: 1, red: 2, black: 3.

Squid munc18 (sSec1), a homolog of the rat protein (munc18a), has been crystallized as a free protein, i.e. unbound to the squid syntaxin [22], and three variations of the structure are available. The following section examines the similarity between the dynamics behavior of the wild-type munc18a during the simulations and the resolved crystal structures of its squid homolog, sSec1. Figure 2 presents a superposition of the three available sSec1 crystal structures (1EPU.pdb, 1FVF.pdb and 1FVH.pdb) and the munc18a crystal structure (3C98.pdb). In the three simulations, domain 3a, and particularly the β-hairpin (residues 263–280) exhibited high structural variability, sampling manifold structures (Figure 2B). Similarly, the three resolved sSec1 structures exhibit high variability among them in the structure of domain 3a. In the simulations, domain 1 remarkably preserved its secondary structure and we observed a clear rotational motion of this domain. Similarly, superposition of the three crystal structures of the squid protein shows that they share the same secondary structure for domain 1, but domain 1 is positioned in a slightly different angle reflecting a rotation motion of this domain (Figure 2C).

### The β-hairpin of domain 3a as a potential gate for release/binding of syntaxin

**Figure 3:**
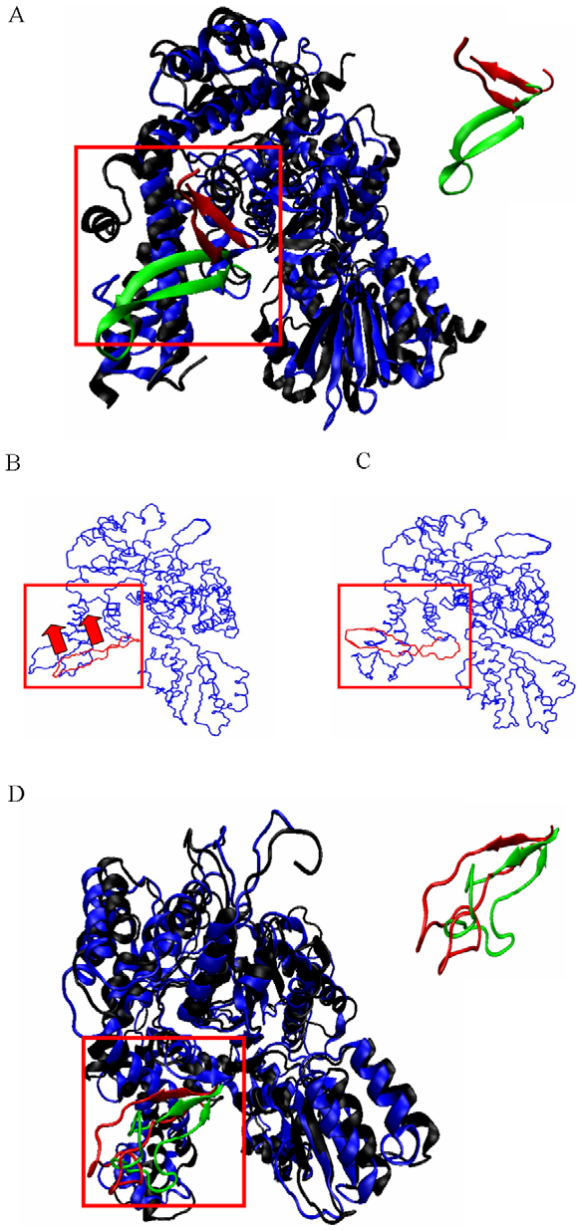
Comparison of munc18a dynamics with the crystal structure of Sly1p, the yeast Golgi homolog. A) Superposition of the munc18a crystal structure taken from its structure in the complex with syntaxin (3C98.pdb, [12]) and the crystal structure of Sly1p, the yeast Sec1/munc18 protein, taken from its crystal structure with the N-peptide of the Golgi syntaxin, Sed5p (1mqs.pdb, [23]). The proteins are presented in ribbon form, in blue and black, respectively. The β-hairpin in domain 3a, present in both of the structures, is marked in green and red, respectively. Inset: The β-hairpin in domain 3a, present in both of the structures, is marked in green and red, respectively. Note the difference in the hairpin's positions in the two structures. B, C) The first eigenvector of simulation 2 exhibits substantial movement of the β-hairpin in domain 3a upwards. B) The first frame of the eigenvector movie (see Methods), representing the starting point of the movement. The red arrows indicate the upward direction of the movement of the β-hairpin during the simulation. C) The last frame of the movie's eigenvector (see Methods), representing the maximum point of the movement. D) Superposition of the Sly1p full-length reconstructed structure at t = 0 (black) and after 15 ns of MD simulation (blue) both presented in the ribbon form. The different positions of the β-hairpin in domain 3a are presented (in red and green respectively). Inset: magnified superposition of the β-hairpin in domain 3a, present in both of the structures (green and red, respectively).

The β-hairpin in domain 3a of munc18a (residues 261–280) plays a prominent role in the interaction of munc18a with syntaxin-1a. Eight amino-acid residues out of the 19 that compose the β-hairpin are engaged in interactions with the H_3_ domain of syntaxin, making the hairpin an essential element in the binding of syntaxin, and in keeping syntaxin in its closed (inactive) structure. Therefore, any fluctuations in the position of the β-hairpin might influence the affinity of syntaxin to munc18a and might cause syntaxin to alternate to its open structure. Comparison of the munc18a structure to the crystal structure of Sly1p, the yeast Golgi homolog of munc18a (1mqs.pdb, downloaded from PDB [23]) indicates that the hairpin of the later, although partially unstructured, resides in a much higher position than in the munc18a crystal structure (Figure 3A). In this structure, the Golgi resident syntaxin (sed5p) is absent from the cavity area and the crystal structure only includes its N-terminal peptide which is bound to the area of domain 1. Interestingly, in simulation 2 of munc18a (Table 1), we traced a prominent motion of the β-hairpin of the protein, moving during the simulation from its original location in the crystal structure outwards and upwards, protruding from the rest of the protein (Figure 3, B and C). This motion, resulting in a position similar to that seen in the crystal structure of the Sly1p, confirms the possibility raised before that the motion of the β-hairpin might serve as a mechanism for the release of syntaxin from the cavity area [22]. To further confirm this notion, we reconstructed the full structure of Sly1p (see Methods) and performed a simulation of its dynamics under the same conditions of the munc18a simulations. We focused on the movement of the β-hairpin in domain 3a (residues 298 to 327) during the simulation. Indeed, during the 20 ns of the simulation, we observed an extensive rotation-translation movement of this region downwards approaching domain 3a (Figure 3D) and consequently narrowing the width of the cavity. Thus, the β-hairpin might serve as a gate for the cavity, opening and closing the cavity when needed. In the current study, we show that this motion can occur spontaneously with no interference from any additional factor(s); although we cannot determine the probability of this type of motion in the free or syntaxin-bound munc18a, these data support the hypothesis that the β-hairpin serves as a switch for syntaxin-binding or unbinding.

### The dynamics of the phosphomimetic mutated munc18a (munc18a^S306D,S313D^)

**Figure 4:**
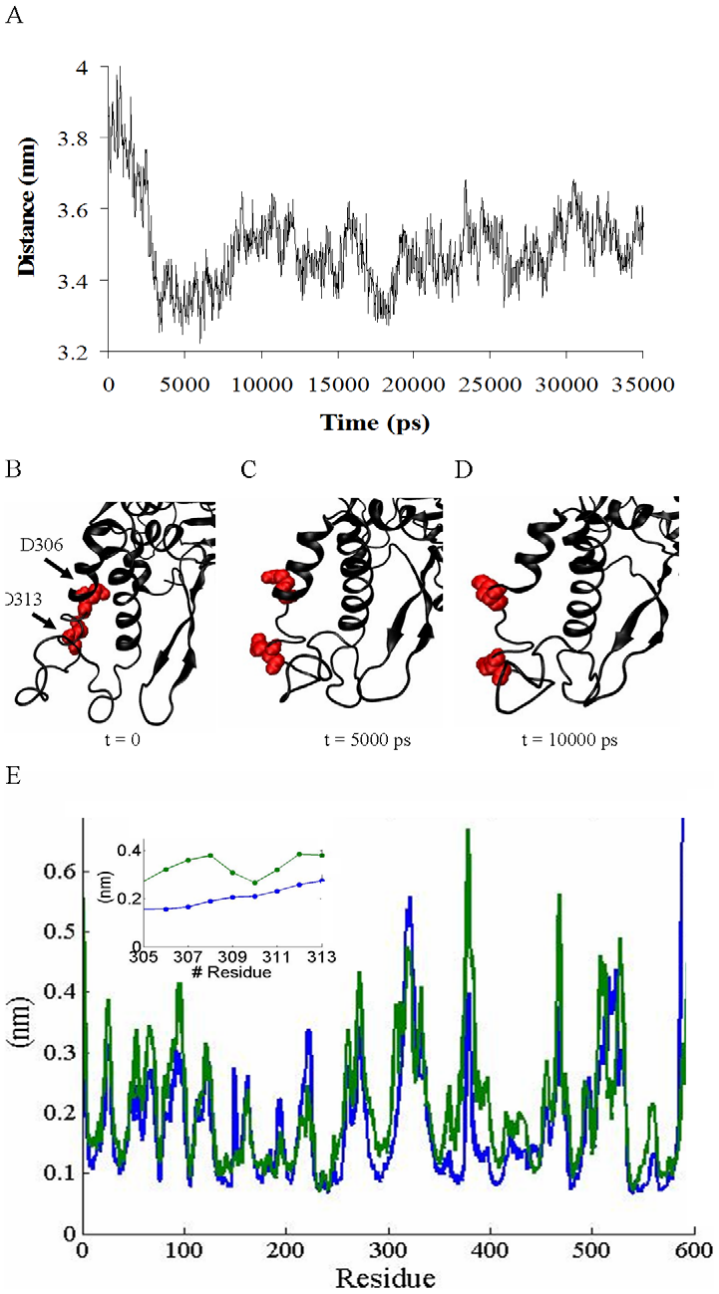
Cavity closure in munc18a^S306D,S313D^ phosphomimetic mutant structure. A) Measurements of the distance between the centers of mass of domains 3a and 1 during the simulation of mutant munc18a^S306D,S313D^. B–D) Structural changes in the positions of the phosphomimetic residues (Glu 306 and Glu 313) of munc18a (simulation M1, Table 1). Snapshots taken from the MD simulation of munc18a^S306D,S313D^; at B) t = 0, C) t = 5000 ps, D) t = 10000 ps. E) Comparison of the RMSF values of munc18a residues in the wild-type (simulation 1) versus the phosphomimetic mutant simulations (blue and green curves, respectively). Inset: magnification of E in the area of the phosphomimetic mutations (residues 305–313), exemplifying the higher RMSF values in this region in the munc18a^S306D,S313D^ simulation.

After characterizing, in detail, the dynamics of the wild-type munc18a, the next step of our study was to examine the dynamic behavior of the phosphomimetic munc18a^S306D,S313D^ and determine the differences compared to the wild-type dynamics. Characterizing the differences in the dynamics of munc18a^S306D,S313D^ will assist to determine a mechanism that might explain the reduced affinity of syntaxin to munc18a^S306D,S313D^/phosphorylated munc18a. Following *in-silico* insertion of the mutations (See Methods) into the wild-type structure, the mutant was simulated under the same conditions as the wild-type (∼35 ns, simulation M1, Table 1). Strikingly, analysis of the fluctuations in the distance between the centers of mass of domains 3a and 1 indicated a marked decrease in the distance between the centers of mass of domains 3a and 1 (Figure 4A). Already in the first ∼3 ns of the simulation, the distance decreased from 3.9 nm to 3.3 nm, and during the rest of the simulation, the distance stabilized (∼3.5 nm) exhibiting only further minor fluctuations (Figure 4A). Observation of the mutant dynamics showed that the decrease in the distance between the centers of mass of domains 3a and 1 represents a process of closure of the cavity between these domains. The closure leads to the preferential stabilization of a distinct closed-cavity conformation of munc18a^S306D,S313D^, a conformation that probably cannot bind syntaxin via the cavity. Further examination of the closure process shows that the conformational change in the protein includes a structural disruption in the area of the mutations, which is located in domain 3a, on the side opposite to the cavity (Figure 4, B–D). Calculation of the average local RMSF (Root Mean Square Fluctuations) in the area of the mutations (residues 306–313) showed a large increment of 28% to 120% in the specific RMSF values of these residues with respect to the wild-type values, indicating substantial movements of this region (Figure 4E). This structural disruption on one side of domain 3a might destabilize its overall structure, allowing the area adjacent to the cavity to move towards domain 1, located on the other side.

### Tracking the cavity closure by ED analysis

**Figure 5:**
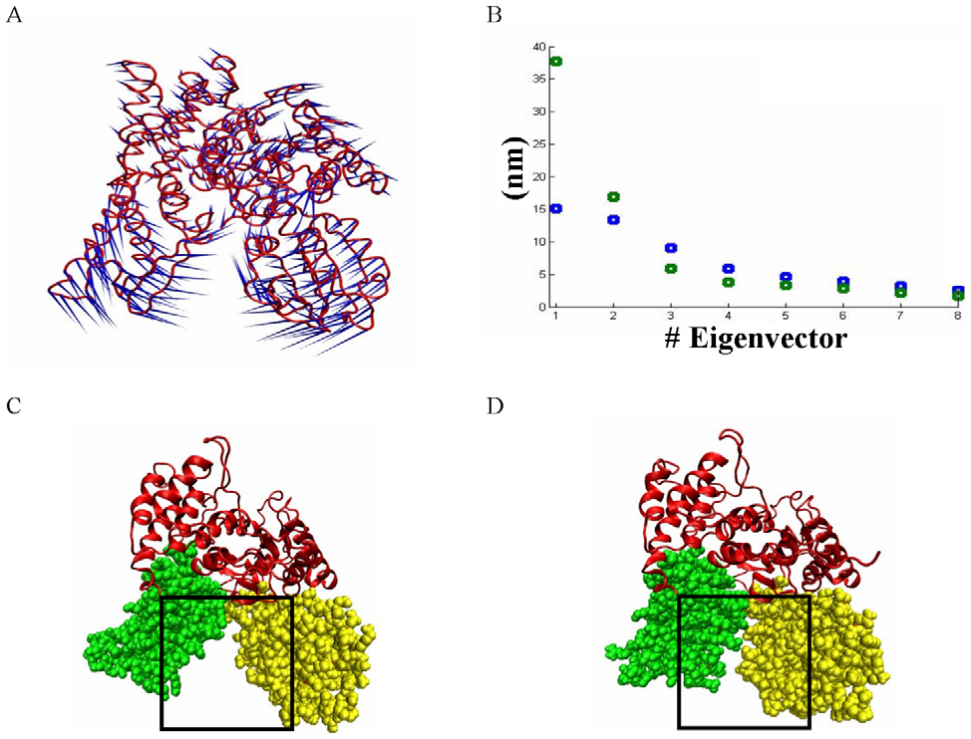
The closure of the cavity as detected by ED analyses of munc18a^S306D, S313D^ simulation. A) Porcupine plot, presenting the first eigenvector in the mutant munc18a^S306D,S313D^ simulation as produced by ED analysis (M1) performed by the Dynatraj tool. B) Comparison of the magnitudes of the eight main eigenvectors of the GROMACS-based ED analysis extracted from the simulations of wild-type munc18a (simulation 1, blue squares) and munc18a^S306D,S313D^ (simulation M1, green squares). C, D) Snapshots of the first (t = 0, C) and the last (t = 35 ns, D) frames of the second munc18a^S306D,S313D^ (Simulation M2, Table 1) simulation.

ED analysis performed both using Dynatraj and by GROMACS for the most dominant motions of the munc18a^S306D,S313D^ simulation (Methods), demonstrated that the closing motion of the cavity (Figure 5A) occurs as part of the most dominant motion in the simulation. The GROMACS-based ED analysis shows that the most dominant motion in the munc18a^S306D,S313D^ simulation is twice the size of the main motion of the wild-type munc18a simulation and encompasses 37% of the total movement of the protein during the simulation (Figure 5B).

To examine whether the phosphomimetic mutations can induce the closure of munc18a cavity also when the structure was already well-relaxed, *In-silico* phosphomimetic mutations were inserted into the well-relaxed structure of munc18a (the structure obtained after 35 ns simulation of the wild-type, see Methods and Table 1), and the structure was simulated from that point for another 35 ns (Simulation M2, Table 1). The phenomenon of cavity closure was clearly reproduced, but the time course of the process was different (data not shown). Comparison of the structures in the first and last frames of this simulation clearly illustrates two distinct conformations: the initial open-cavity conformation and the final closed-cavity conformation (Figure 5, C and D). For comparison to the wild-type simulation (Figure 1E), the change in the distance between residues Gly 26 in domain 1 and Glu 273 in domain 3a, during this munc18a^S306D, S313D^ simulation, is presented. The distance between these residues decreased during the last 20 ns of the simulation (Figure S3) demonstrating again the closure of the cavity. In addition, a video of the trajectory of this simulation, demonstrating the closure process is presented (Video S1).

### The closed-cavity conformation is the dominant structure of the phosphomimetic mutant munc18a^S306D, S313D^

To determine the relative stability of the structures that munc18a^S306D,S313D^ samples during the simulation, and to identify the most stable and dominant structure in the mutant simulations, we had used another quantitative analysis tool for the simulations termed, cluster analysis ([24], Methods). Briefly, Cluster analysis segments the structures that the protein samples during the simulation into sub-groups (termed, clusters). The structures are divided to clusters according to an adjustable RMSD (Root Mean Square Deviations) cut-off value that defines the variance between structures that populate the same cluster ([24], Methods). Comparison of the cluster analyses performed for the phosphomimetic munc18a^S306D,S313D^ and the wild-type munc18a simulations, using the same RMSD cut-off value, showed that munc18a^S306D,S313D^ samples fewer conformations than the wild-type during the simulations, having less distinct clusters, 48 vs. 72 respectively (Table 2). Moreover, the three largest clusters in the munc18a^S306D,S313D^ simulation encompass about 27% of the total structures population compared with only 16% as determined in the wild-type munc18a cluster analysis. Analyzing the size of the syntaxin-binding cavity in the three largest clusters of the munc18a^S306D,S313D^ compared to the wild-type shows that the three largest clusters in the phosphomimetic munc18a^S306D,S313D^ simulation demonstrated a smaller variance in the values of the distances between the centers of mass of domains 3a and 1 exemplifying that the size of the cavity is relatively unchanged compared to the size of the cavity in the wild-type three main clusters. As detailed in Table 2, the mean distance between the centers of mass of the two domains is significantly shorter for the phosphomimetic munc18a^S306D,S313D^ illustrating that a closed-cavity conformation predominates in the phosphomimetic munc18a^S306D,S313D^ three largest clusters (Table 2). In summary, the cluster analysis shows that munc18a^S306D,S313D^ samples less distinct conformations during the simulations. The mutant is more rigid in the cavity's region than in the wild-type simulation and a closed-cavity conformation predominates.

**Table 2:** Summary of the results from the cluster analysis of the simulations of wild-type munc18a and munc18a^S306D,S313D^ (dt = 10 ps).

Condition	Number of clusters	Number of clusters≥1% of ∑structures	% of largest three clusters out of ∑structures	# Cluster	Munc18a (1) mean distance	Munc18a^S306D, S313D^ (M1) mean distance
Munc18a WT (n = 3)	72 (3)	34 (3)	16.3 (3)	1	3.8 (0.1)	3.52 (0.05)
Munc18a^S306D,S313D^ (n = 2)	48 (5)	25 (1)	27.15 (3)	2	3.7 (0.06)	3.48 (0.06)
				3	3.9 (0.1)	3.39 (0.06)

Cluster analysis segments the structures that the protein samples during the simulation into sub-groups according to an adjustable RMSD cut-off value that reflects the extent of similarity between the structures. The analysis was performed using the Gromos algorithm (RMSD cut-off of 0.2 nm). The table presents the average total number of clusters, the number of clusters that comprise more than 1% of the total number of structures, the percentage of the three main clusters out of the total number of clusters, and the mean distance between the centers of mass of domains 3a and 1 together with the STDEV in the three largest clusters of simulations 1 and M1.

### The stabilization of the closed-cavity conformation by inter-domain hydrogen bonds

**Figure 6:**
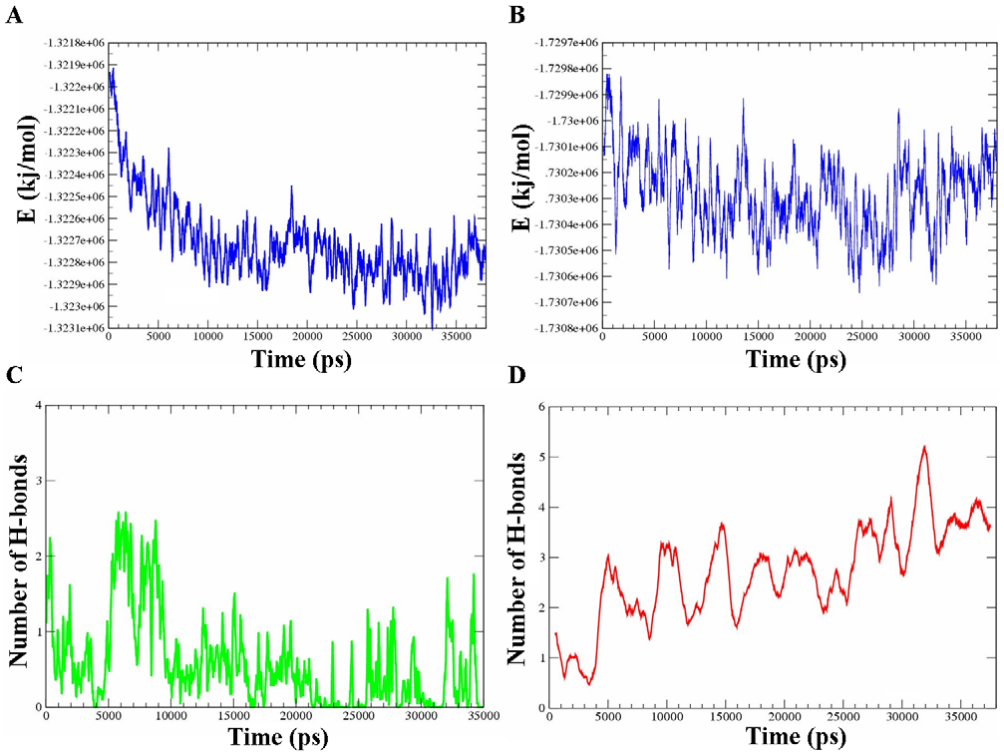
Energetic stabilization is correlated to munc18a^S306D,S313D^ closure of the cavity. A) Averaged total energy changes during the simulation of the munc18a^S306D,S313D^ (simulation M1). B) Time-dependent changes in the Coulomb energy component. C) Time-dependent change in the number of hydrogen bonds between domains 3a and 1 in the simulation of the wild-type munc18a. D) Hydrogen-bond formation between domains 3a and 1 during the simulation of the munc18a^S306D,S313D^ (M1).

**Figure 7:**
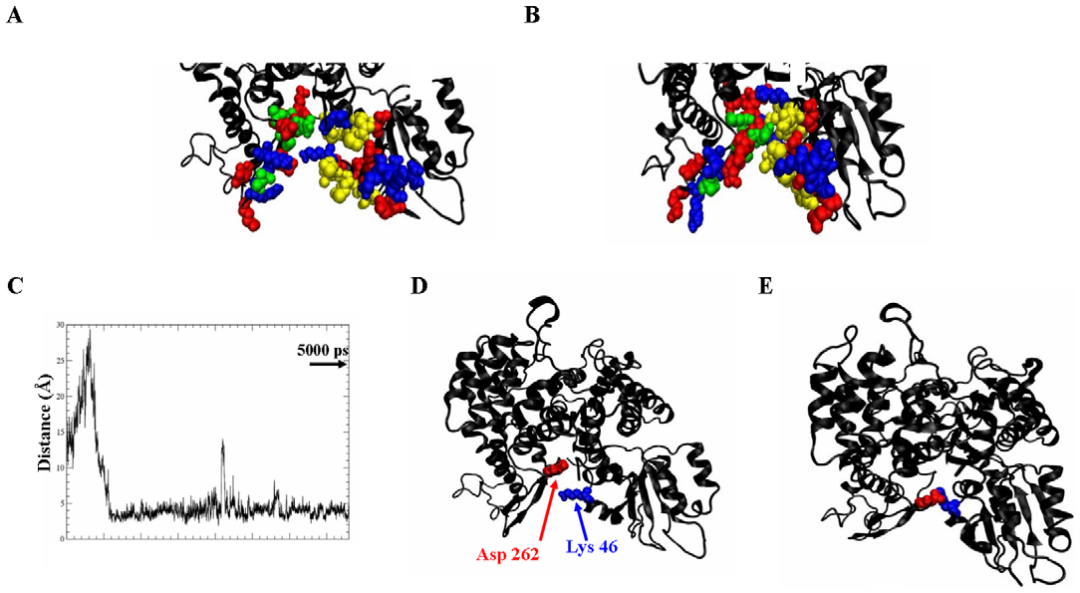
Electrostatic and LJ interactions of residues on either side of the munc18a cavity stabilize its closure. A, B) Snapshots taken from the mutant munc18a^S306D,S313D^ simulation; Munc18a^S306D,S313D^ charged residues are presented in the space-fill model (blue, positive residues and red, negative residues). A) t = 0, B) t = ∼37 ns. Hydrophobic interactions further stabilize the closure of the cavity; hydrophobic residues in domain 1 (green) and in domain 3a (yellow). C) The distance between Lys 46 and Asp 262 during the ∼37-ns simulation (dt = 10 ps). D, E) snapshots taken from the simulation showing the position of Lys 46 and Asp 262 at D) t = 0, E) t = ∼37 ns.

In order to identify the driving force for the cavity closure process, we examined the energetic components (Lennard-Jones [LJ] and electrostatic) of munc18a^S306D,S313D^ during the simulation time. Inspection of the change in the energetic components of munc18a^S306D,S313D^ shows that the closing motion of the protein was correlated with a decrement in the sum of the electrostatic and LJ energy components of the system indicating stabilization of the structure (Figure 6, A and B), and the formation of extra electrostatic and hydrophobic interactions. Specifically in the cavity area, we monitored the time-dependent pattern of hydrogen bonds and found that three to five additional hydrogen bonds were formed during the simulations between residues located on both sides of the cavity (Figure 6D). The later is in contrast to the wild-type simulation, that during the same simulation time, the number of hydrogen bonds between domains 3a and 1 fluctuated between 0–2 (Figure 6C). The interactions between residues located on both sides of the cavity kept them in proximity and stabilized the closed-cavity conformation. In addition to the Hydrogen bonds that were formed during the munc18a^S306D,S313D^ simulation, LJ interactions between residues in domains 3a and 1 further stabilized the closed state (Figure 6B and Figure 7, A and B, residues in green and yellow). Table 3 summarizes the interactions observed during the simulation between residues located on both sides of the cavity.

**Table 3:** A list of pairs of residues, located on the sides of the cavity, either in domain 1 (right side) or domain 3a (left side), that interacted stably with each other during the munc18a^S306D,S313D^ simulations (M1 and/or M2), stabilizing the closure of the cavity.

Residues in domain 1	Residues in domain 3a
Met 38	Asn 261
Arg 39	Tyr 254/Glu 283
Ser 42	Asp 262
Ser 43	Glu 283
Lys 46	Asp 262/Leu 281

It should be noted that Arg39 [9] and other munc18a residues that are essential for its interaction with syntaxin, were found to be involved in the inter-domain interactions, bringing both sides of the cavity together.

Similarly, Lys46 (domain 1) that is involved in the interaction with syntaxin forms an electrostatic interaction during the free munc18a^S306D,S313D^ simulation (M2) with Asp262 located in domains 3a. Figure 7C depicts the decrease in the distance between Lys46 and Asp262 to ∼2.5 nm, as they approach each other during the simulation, forming a stable electrostatic interaction already after 5 ns of the simulation (Figure 7, C–E). The analysis of the energetic components of the munc18a^S306D,S313D^ system during the simulation shows that the mutant protein (munc18a^S306D,S313D^) is energetically-stabilized in the closed-cavity conformation in which residues on both sides of the cavity interact with each other. Therefore, the binding of syntaxin to munc18a^S306D,S313D^ requires breaking several intra-molecular electrostatic bonds and as a result might become energetically unfavorable.

### The closed-cavity conformation is induced specifically by the phosphomimetic mutations

**Figure 8:**
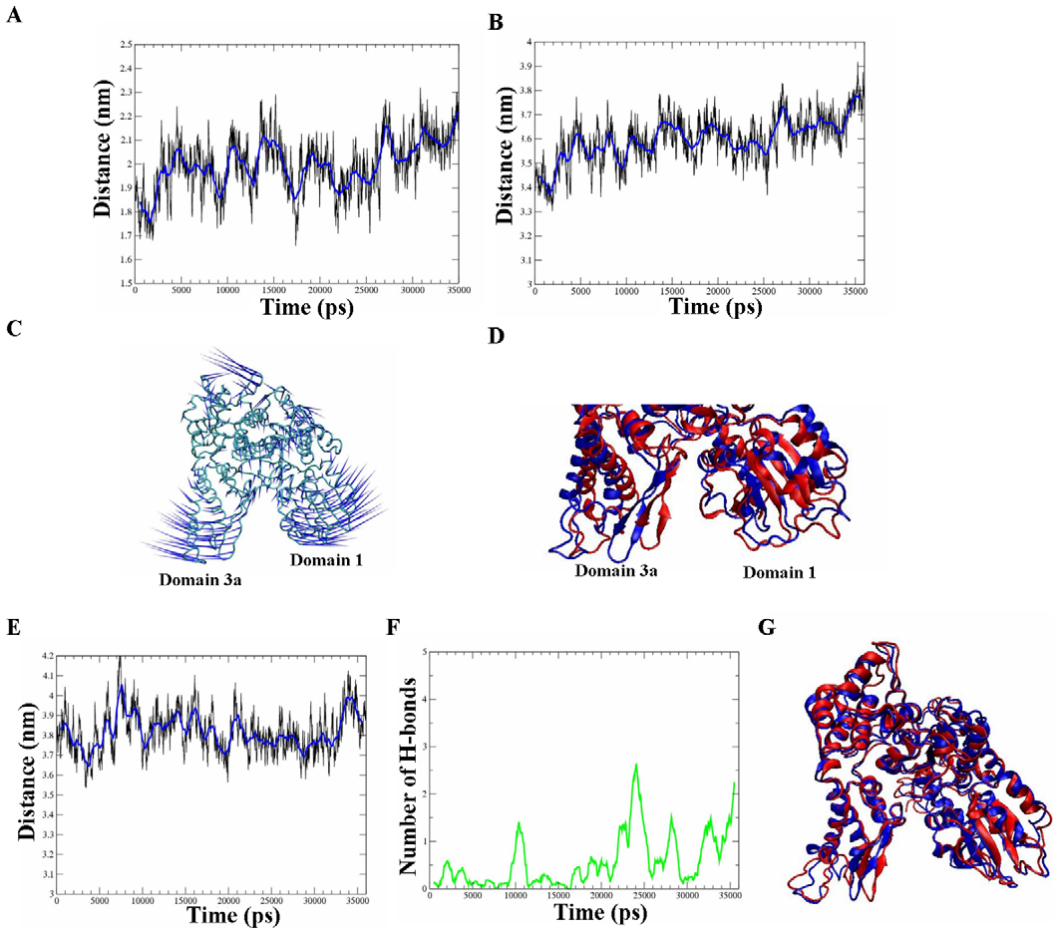
Back-mutations in munc18a restore its dynamic nature and induce gradual reopening of its cavity. A) Measurement of the distance between the centers of mass of domains 3a and 1 during back-mutated wild-type munc18a simulation (munc18a^D306S,D313S^, 36 ns). B) The distance of the centers of mass of two regions adjacent to the cavity: residues 35–70 (domain 1) and residues 260–280 (domain 3a). C) Porcupine plot demonstrating the opening motion of the cavity (the fourth most dominant eigenvector of the protein in the simulation). D) Superposition of domains 3a and 1 from the last frames in the simulations of munc18a^S306D,S313D^ (t = 35 ns, red) and munc18a^D306S,D313S^ (t = 36 ns, blue). E) Measurement of the distance between the centers of mass of domains 3a and 1 during the munc18a^S306A,S313A^ simulation (36 ns). F) Time-dependent change in the number of hydrogen bonds between domains 3a and 1 in the munc18a^S306A,S313A^ simulation G) Superposition of the structure of munc18a^S306A,S313A^ in the first (t = 0, red) and last frame (t = 36 ns, blue) of the simulation.

We next investigated whether the closed-cavity conformation is reversible, whether it is induced directly by the insertion of the phosphomimetic mutations and whether the protein can regain its flexibility in the area of the cavity. We removed the phosphomimetic mutations from the structure of the protein in the last frame of the munc18a^S306D,S313D^ simulation and performed another simulation of 36 ns of this structure mutated back to the wild-type (D306S, D313S). This simulation showed that the back-mutated wild-type protein gradually regains its dynamic nature in the cavity area and the cavity starts to reopen (Figure 8, A and B). The distance between the centers of mass of two regions adjacent to the cavity: residues 35–70 (domain 1) and residues 260–280 (domain 3a) increased from 1.8 nm to 2.2 nm during the 36-ns back-mutation simulation (munc18a^D306S, D313S^, Figure 8A). Next, we extended this analysis by looking at the relative motion of larger sections of the protein; measurement of the distance between the centers of mass of domains 3a and 1 during the munc18a^D306S, D313S^ simulation indicated that the distance gradually increases from 3.4 nm up to 3.8 nm, reflecting reopening of the cavity. The opening movement of the cavity was also observed by ED analysis; in Figure 8C, we present a porcupine plot of the fourth eigenvector of the dynamics demonstrating by the direction and length of the ‘needles’ a clear expansion of the cavity. Finally, a straightforward superposition of domains 3a and 1 from the last frames in the simulations of munc18a^S306D,S313D^ (t = 35 ns, red) and munc18a^D306S,D313S^ (t = 36 ns, blue) indicates that the positions of domains 3a and 1 are further away from each other in the back-mutated munc18a^D306S, D313S^ compared to the phosphomimetic munc18a^S306D,S313D^ and similarly to the wild-type (Figure 8D). The results suggest that the phosphorylation/phosphomimetic mutations induce a closed-cavity conformation that can be reversed upon dephosphorylation/back-mutations of the protein.

Mutations of Ser 306 and Ser 313 to Ala in munc18a were shown to turn the protein to be non-phosphorylated and had no affect on syntaxin binding [14], [25]. To check the specificity of the closed-cavity conformation to the phosphomimetic mutations in these positions, another simulation (36ns long) was performed, following the dynamics of the non-phosphorylated mutant munc18a^S306A,S313A^ under the same conditions as in the previous simulations. Measurement of the distance between the centers of mass of domains 3a and 1 during the munc18a^S306A,S313A^ simulation shows that the distance was fluctuating between 3.5 to 4.2 nm (Figure 8E), similarly to the fluctuations that were observed in the wild-type simulation (Figure 1C). We did not track any substantial movement of domains 3a and 1 towards each other, thus, no closure of the cavity was observed as was depicted in the phosphomimetic munc18a^S306D,S313D^ simulations. Analysis of the time-dependent change in the number of hydrogen bonds between domains 3a and 1 shows that the number of hydrogen interactions remained 0 or 1 during most frames of the simulation indicating that no new hydrogen bonds were formed during the simulation between residues located in domains 3a and 1, in contrast to the phosphomimetic munc18a^S306D,S313D^ simulations (Figure 8F). In summary, the mutant munc18a^S306A,S313A^ did not adopt a closed-cavity conformation (Figure 8G) and the dynamics resembled that of the wild-type state (Figure 8, E–G) indicating specificity of the closing phenomenon to the phosphomimetic mutations in these positions.

## Discussion

The current study reveals, for the first time, new conformations that munc18a can adopt when it is unbounded to syntaxin. Based on a rigorous analysis of a comprehensive set of molecular dynamics simulations we were able to monitor the dynamics of the wild-type free munc18a in comparison to its mutant forms (phosphomimetic, back-mutated and non-phosphorylated mutants), focusing on the structural changes that occur during the trajectories in the main syntaxin-binding site, the cavity between domains 3a and 1. We show that munc18a, in its syntaxin-unbounded form, is in a dynamic equilibrium between conformations varying in the size of its syntaxin-binding cavity located between domains 3a and 1.

Specifically, we found that munc18a can adopt a stable conformation where its cavity, serving as the main syntaxin-binding site, is mostly blocked by inter-domain interactions. This conformation is induced following *in silico* insertion of phosphomimetic mutations in positions 306 and 313 (S306D, S313D). We propose that the observed reduction in affinity of munc18a to syntaxin following phosphorylation or insertion of phosphomiemtic mutations as shown experimentally is a result of preferential stabilization of a conformation of munc18a where the syntaxin-binding site is less accessible for syntaxin. This conformation of munc18a makes the binding of syntaxin less probable, and energetically and sterically unfavorable.

Our proposed mechanistic explanation is supported by a few studies carried out in the past that already speculated that munc18a might have additional distinct conformations different from the one that was resolved (bound to syntaxin) in the published crystal structure [9], [12]. Previous studies suggested that the munc18a conformations could be induced by interactions with other proteins, such as Rab, Rab effector or munc13 [9].

However, to the best of our knowledge, there is no other available resolved conformation (i.e crystal structure) of munc18a.

Another indication for the existence of several munc18a conformations is the putative binding site of the protein cyclin-dependent kinase 5 (CDK5) in munc18a. CDK5 has been shown to phosphorylate munc18a and to mediate the disassembly of the munc18a-syntaxin-1a complex, with the assistance of other proteins. The site of CDK5-mediated phosphorylation in munc18a is located between domains 2 and 3 (residue Thr574). In the crystal structure of the munc18a-syntaxin complex, this region of munc18a is buried in the protein and therefore inaccessible, indicating that CDK5 probably interacts with a different conformation of munc18a that was not determined yet [9], [21].

**Figure 9:**
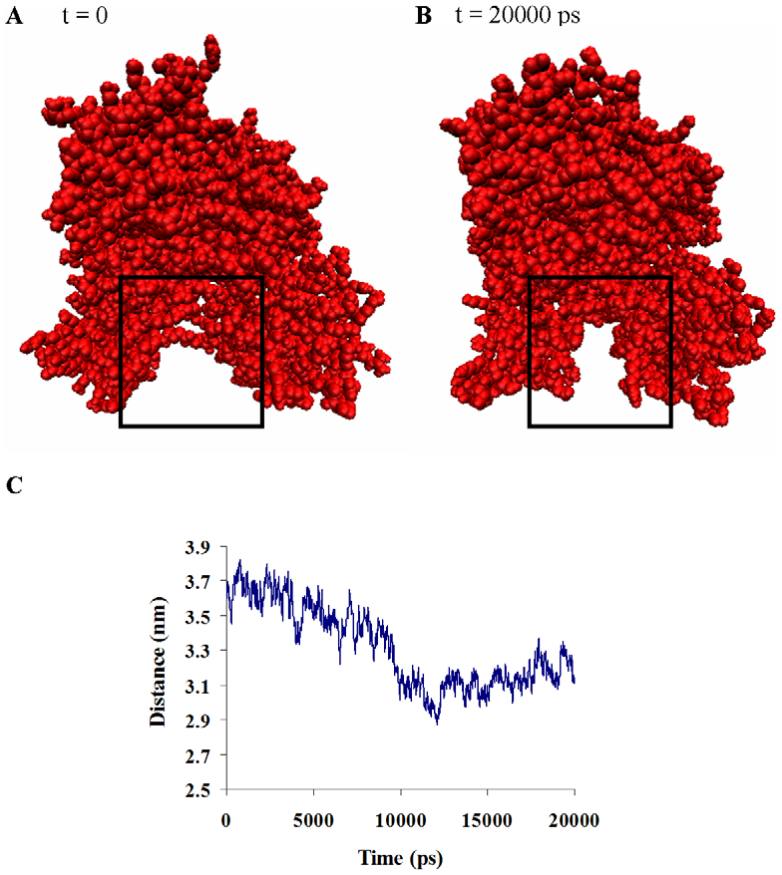
Munc18a^F115E^ adopts a closed-cavity conformation. Two snapshots taken from a 20-ns-simulation of munc18a^F115E^ demonstrate the closure of the cavity between domains 3a and 1 during the simulation of this mutant as well. A) t = 0, B) t = 20 ns, the cavity area is framed in both A and B. C) Measurement of the distance between the centers of mass of domains 3a and 1 during the Munc18a^F115E^ simulation.

The closed-cavity conformation of munc18a is specifically induced by the phosphomiemetic mutations; however it is not exclusively present in this mutated form of the protein. Molecular dynamics simulation of another mutated form of munc18a - munc18a^F115E^ [13] showed that the introduction of this mutation induced closure of the cavity as well (Figure 9, A–B). The closure was initiated by a dominant movement of domain 1 towards domain 3a. These results suggest that the closed-cavity conformation can be driven by several types of mutations. The tendency of the protein to form this conformation might be a general mechanism explaining the impaired binding of several mutated forms of munc18a to syntaxin.

The established hypothesis attributes the reduced affinity of the phosphorylated munc18a (or the phosphomimetic mutant munc18a^S306D,S313D^) to syntaxin to the local repulsion of syntaxin by the negative charges of the phosphates (or glutamates) in this region of munc18. This repulsion was suggested to reduce the compatibility and the overall affinity of the complex [14]. The hypothesis presented in the current study, based on extensive molecular dynamics simulation and analyses, challenges this paradigm and suggests that the reduced affinity results from closure of the cavity of munc18a, making it inaccessible for syntaxin binding in this area.

Many key biological processes such as the synaptic processes [21], [26] are regulated by protein phosphorylation. In order to understand the effects of this process, it is essential to characterize specifically the structural changes induced by phosphorylation, leading to a change in the affinity of proteins to target proteins. In this study, we followed structural changes that phosphorylation might induce and we were able to provide a novel mechanism for explaining experimental results showing reduced affinity between proteins. As the potential of phosphorylation to induce substantial conformational changes in proteins was already shown in previous studies [27], [28], [29], [30], we suggest that the present conventional paradigm, explaining the reduced affinity of the phosphorylated munc18a to syntaxin as merely a local repulsive phenomenon, is rather simplified. Efforts should be aimed at tracking the global dynamic conformational changes that occur in the phosphorylated munc18a or in other mutated forms of munc18a in attempt to resolve munc18a conformations and in particular the closed-cavity conformation.

## Methods

### Molecular dynamics simulations of munc18a

All simulations performed were using the coordinates of munc18a crystal structure that were taken from the recently refined crystal structure of the syntaxin-1a-munc18a complex, determined by x-ray crystallography at a resolution of 2.6 Å [9], [12]. The crystal structure coordinates, taken from the Protein Data Bank (PDB file: 3C98.pdb), include 556 residues out of the 594 residues of the full sequence of munc18a: 6 residues (317–323) in domain 3a and 25 residues (506–531) in domain 2 have not been structurally resolved. In addition, at both terminals; the first three residues of the N-terminal (amino-acid residues 1–3) and residues 593–594 of the C terminal were not resolved as well. Three simulations of wild-type munc18a were performed differing in the tools used for completion and structural prediction of the missing regions. In simulations 1 and 2, the missing residues were added to the structure and modeled using the Swiss-PDB program ([16], http://www.expasy.org/spdbv/) and in simulation 3, the Rosetta software [17] was used for the completion and modeling as detailed below. In the Swiss-PDB, an energy-minimizing computation was performed by the Swiss-PDB tool using Gromos96 implementation of the Swiss-PDBViewer following the addition of the residues.

All MD simulations presented in this article were performed using the GROMACS 4.0 suite of software [31], using the GROMACS 53a6 force field [32]. The protein was embedded in a dodecahedron box containing the SPC water molecules [35,097 molecules for the Swiss-PDB based structures (1 and 2) and 34,972 molecules for the Rosetta-based structure (simulation 3) that was extended to at least 15 Å between the protein's structure and the edge of the box. Assuming normal charge states of ionizeable groups corresponding to pH 7, the net charge of munc18a structure is −4e. Hence, 74 sodium and 70 chloride ions were added to the Swiss-PDB structure trajectory box at random positions, to neutralize the system at a physiological salt concentration of 100 mM. Similarly, 73 sodium ions and 69 chloride ions were added to the Rosetta structure trajectory box (simulation 3). The difference in ion numbers is a result of the difference in the number of water molecules. Prior to the dynamics trajectory, internal constraints were relaxed by energy minimization. Following this step, an MD equilibration run was performed under position restraints for 40 ps. Then, unrestrained MD runs were initiated. Two runs of 35 ns each were performed for the Swiss-PDB structure (simulations 1 and 2) and a single run of ∼35 ns for the selected Rosetta structure (simulation 3). During the MD runs, the LINCS algorithm [33] was used in order to constrain the lengths of all bonds; the water molecules were restrained using the SETTLE algorithm. The time step for the simulation was 2 fs. The simulation was run under NPT conditions, using the Berendsen coupling algorithm to keep the temperature and pressure constant (*P* = 1 bar; *τ_P_* = 0.5 ps; *τ_T_* = 0.1 ps; *T* = 300 K). Van der Waals (VDW) forces were treated using a cut-off of 12 Å. Long-range electrostatic forces were treated using the PME method. The coordinates were saved every 1 ps. Low-pass frequency filtering was performed on the simulations using the g_filter tool of GROMACS.

### Sly1p structure prediction and simulation

The amino acid sequence of the protein Sly1p was fed into I-TASSER (iterative threading assembly refinement algorithm), a 3D protein structure prediction tool [34], [35], [36] in order to predict the full length structure of the protein (671 residues). A partial structure of Sly1p-Sed5p complex crystal structure is available as well (1mqs.pdb, [23]). One of the best-scored Sly1p model structure obtained by the I-TASSER was chosen as the starting coordinates for the Sly1p MD simulation. The simulation was run for 15 ns under the same conditions and procedure as described for the munc18a.

### The mutant munc18a simulations

Simulations of the phosphomimetic double-mutant munc18a^S306D,S313D^ were carried out using the same procedure as described for the wild-type simulations. The Swiss-PDB software was used for *in silico* replacement of Ser 306 and Ser 313 with glutamates. The positions of the mutated residues were optimized and the overall structure was subjected to energy minimization performed by the Swiss-PDB software and then by the GROMACS suite. More details regarding the simulations can be found in Table 1.

The simulations of the back-mutated munc18a (munc18a^D306S, D313S^), the non-phosphorylated munc18a^S306A,S313A^ and of munc18a^F115E^ were performed in the same procedure described above.

### Structure prediction using Rosetta software

The Rosetta program [17] was used to model the missing regions in the crystal structure of munc18a using the loop modeling option as described in details in several studies [18], [37], [38]. Repeated runs of the full-length structure were performed generating a total of 1050 plausible structures. The structures were all scored according to their energy and the structure with the lowest score, representing the most probable structure, was chosen for the MD simulation, termed 3.

### Essential dynamics (ED) analysis

The required covariance matrix and eigenvectors for the ED analysis were obtained by applying the g_covar program of the GROMACS 4.0 package. The analysis was performed on the backbone of the protein. The trajectory was filtered using the GROMACS g_filter program. Movies of 1000 frames representing the pathway between the minimum and maximum points of the movement in the main eigenvectors of each trajectory were formed using the g_anaeig command.

ED analysis was also performed using the Dynatraj tool which is a part of the Dynamite server (http://dynamite.biop.ox.ac.uk/dynamite, [39]). Porcupine plots, to visualize the modes of motion taken from the simulations were produced using the Dynatraj tool [40].

### Cluster analysis

Cluster analysis was performed for the simulations of wild-type and phosphomimetic mutant munc18a^S306D,S313D^ by the command g_cluster of the GROMACS 4.0 package. The cluster analysis was performed using the Gromos algorithm with an RMSD cut-off value of 0.2 nm [24].

## Supporting Information

Figure S1 Similar structural stability of wild-type munc18a in three different simulations.
(0.79 MB TIF)

Figure S2 RMSF and secondary-structure maps of munc18a wild-type structures.
(1.05 MB TIF)

Figure S3 The distance between residues Gly 26 (domain 1) and Glu 273 (domain 3a), residing at either sides of the cavity (calculated for simulation M2).
(0.33 MB TIF)

Text S1 Supplementary information for the article.
(0.03 MB DOC)

Video S1 The closure of the munc18a^S306D,S313D^ cavity. A video of one of the two munc18a^S306D,S313D^ simulations (simulation M2) demonstrating the closure of the protein's cavity.
(6.94 MB MPG)
