# Supplementary figures and images for: Dynamic Conformational Changes in MUNC18 Prevent Syntaxin Binding
Source: PLoS Comput Biol. 2011 Mar 3;7(3):e1001097. doi: 10.1371/journal.pcbi.1001097 (PMC3048386; doi:10.1371/journal.pcbi.1001097)

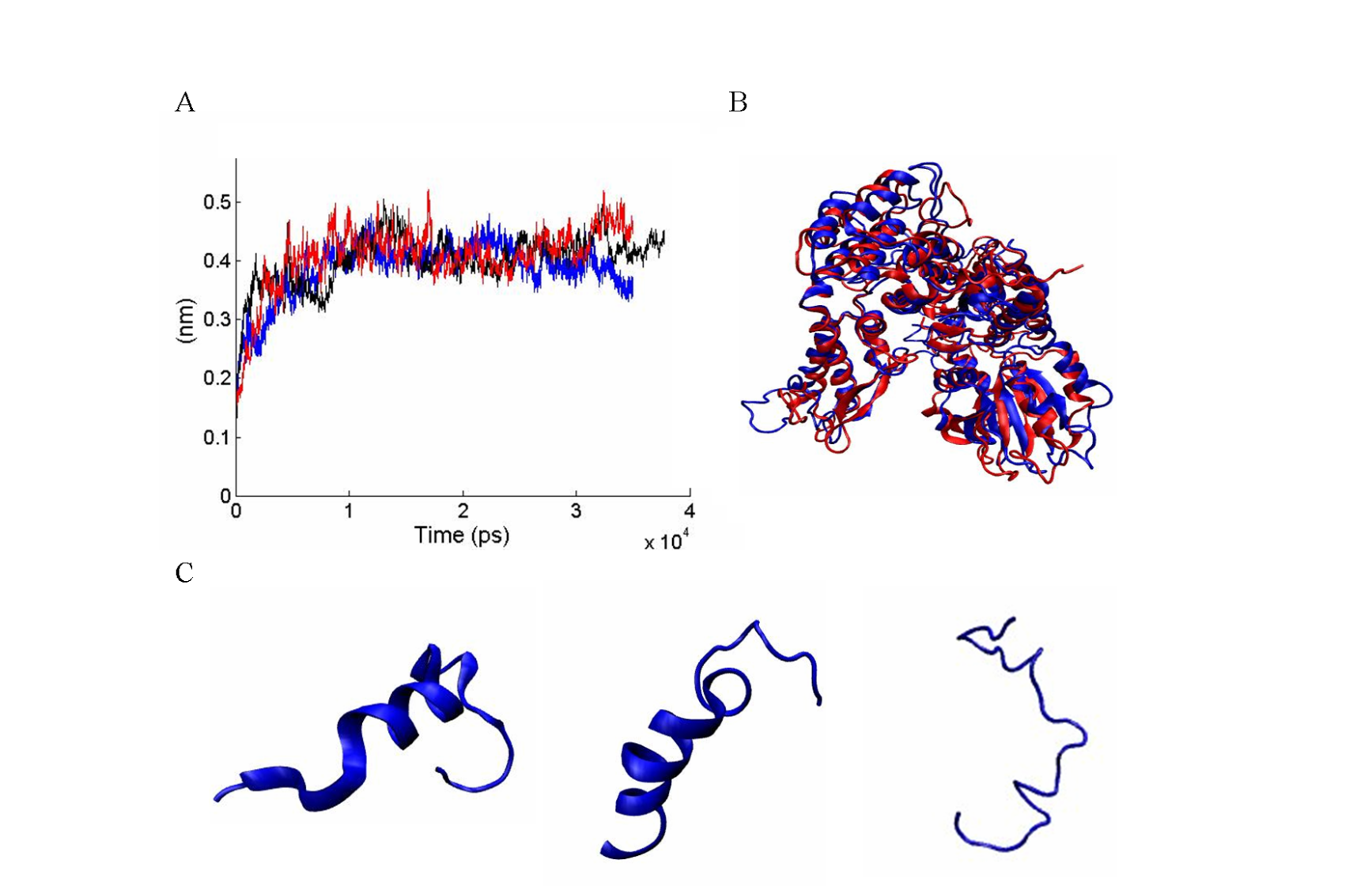

Supplement: Figure S1 — Similar structural stability of wild-type munc18a in three different simulations. (0.79 MB TIF) [file pcbi.1001097.s001.tif]

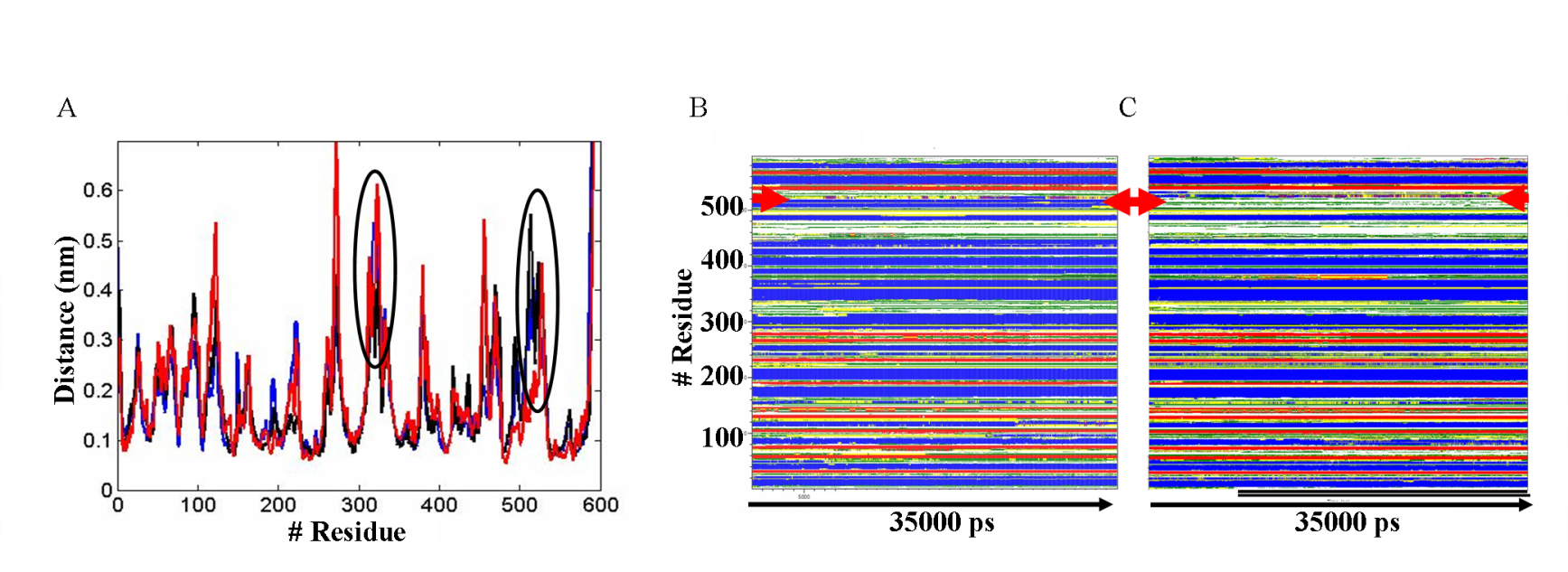

Supplement: Figure S2 — RMSF and secondary-structure maps of munc18a wild-type structures. (1.05 MB TIF) [file pcbi.1001097.s002.tif]

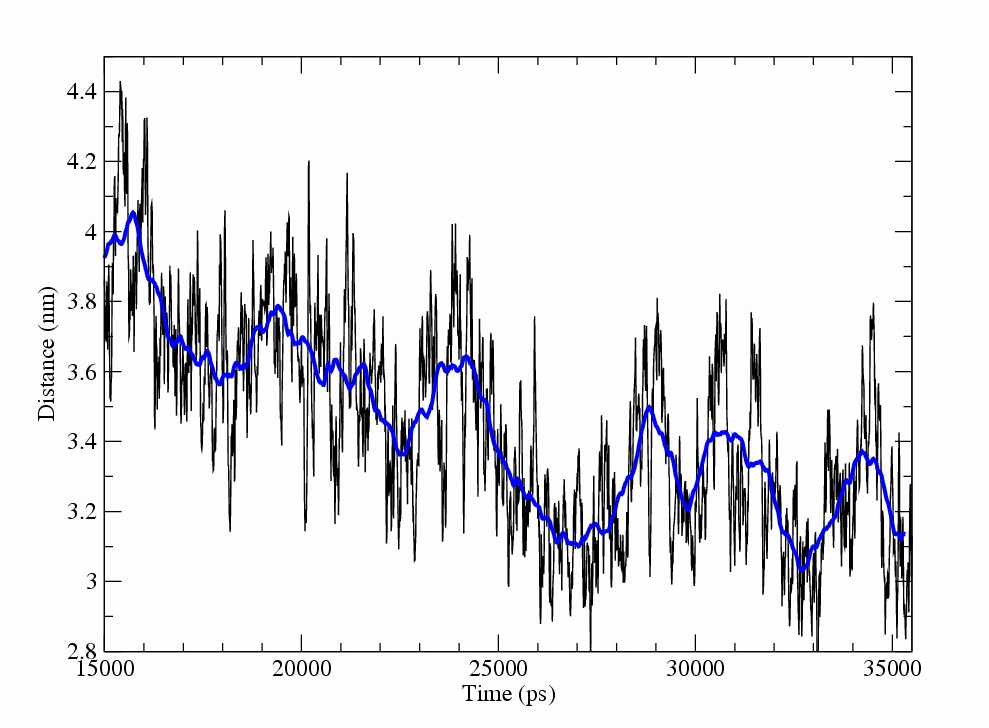

Supplement: Figure S3 — The distance between residues Gly 26 (domain 1) and Glu 273 (domain 3a), residing at either sides of the cavity (calculated for simulation M2). (0.33 MB TIF) [file pcbi.1001097.s003.tif]
