# Supplementary material for: Dynamic Conformational Changes in MUNC18 Prevent Syntaxin Binding
Source: PLoS Comput Biol. 2011 Mar 3;7(3):e1001097. doi: 10.1371/journal.pcbi.1001097 (PMC3048386; doi:10.1371/journal.pcbi.1001097)
Supplement: Text S1 — Supplementary information for the article. (0.03 MB DOC) [file pcbi.1001097.s004.doc]

Text S1

**Munc18a dynamics is independent of the structure of its reconstructed missing regions**

Three molecular dynamics (MD) simulations were performed using the nSec1/munc18a wild-type protein structure taken from the recently published refined crystal structure of the complex of munc18a with syntaxin [1] (3C98.pdb). The munc18a crystal structure was published with two large regions missing from the resolved structure in domain 2 and sub-domain 3a of the protein: residues 317-323 and residues 506-531, respectively. The missing regions were either completed by the Swiss-PDB tool or completed and modeled by Rosetta software as described in the Methods section. We performed three simulations (simulations 1-3, Table 1), which shared similar Cα atom RMSDs (Root Mean Square Deviations) from the initial conformations (Figure S1). This suggested similar behavior of the structures during the simulations, independent of the different methods used for insertion of the missing regions.

A more accurate evaluation of the effects of the method used to complete the missing sections can be gained by comparing the RMSFs (Root Mean Square Fluctuations) of each of the residues. The RMSFs calculated for the three simulations were very similar and the deviations were mostly concentrated in the two regions with the added protein parts, marked in the figure by ellipses (Figure S1, A). Visual inspection of snapshots taken at the end point of each of the three simulations (Figure S2, B) revealed that even though the added parts of the protein adopted somewhat different local structures during the MD simulations (Figure S2, C), the protein retained its overall structure (Figure S2, B). A detailed analysis of the secondary structure of the protein is presented in Figure S1, B and C, each amino acid residue in the protein is assigned a color representing its secondary structure, and the maps presented in Figure S1, B and C show the time-dependent changes in the local elements of the secondary structure. Comparison of the two maps (Figure S1, B and C, corresponding to simulations 1 and 3, respectively, see Table 1) reveals very high resemblance of the secondary-structure patterns. It should be stressed that the two inserted regions (pointed out by red arrows) exhibit a non-identical secondary-structure pattern: the simulation based on the Swiss-PDB exhibits a helical pattern while the Rosetta-based structure simulation exhibits a random coiled one (Figure S1, C). In summary, the reconstructed protein exhibited structural fluctuations that were insensitive to the method used for completing and modeling the missing regions. This suggests that the added regions are highly flexible and that their motion is not tightly coupled with the other modes of the protein.

Our finding that the structural fluctuations of munc18a are not affected by the structure of the added regions (residues 317-323 and 505-531) enables evaluation of the relative motions of the various domains of the protein in the wild-type simulations and their attribution to protein activity and function.

References

1. Burkhardt P, Hattendorf DA, Weis WI, Fasshauer D (2008) Munc18a controls SNARE assembly through its interaction with the syntaxin N-peptide. EMBO J 27: 923-933.
